# MiR-30c-5p loss-induced PELI1 accumulation regulates cell proliferation and migration via activating PI3K/AKT pathway in papillary thyroid carcinoma

**DOI:** 10.1186/s12967-021-03226-1

**Published:** 2022-01-06

**Authors:** Tingting Zheng, Youxing Zhou, Xiaowei Xu, Xin Qi, Jiameng Liu, Yanan Pu, Shan Zhang, Xuerong Gao, Xinkai Luo, Mei Li, Xuefeng Wang, Liyang Dong, Ying Wang, Chaoming Mao

**Affiliations:** 1 Department of Nuclear Medicine, The Affiliated Hospital of Jiangsu University, Zhenjiang, 212000 Jiangsu People’s Republic of China; 2 Department of Surgery, Jiangyuan Hospital Affiliated To Jiangsu Institute of Nuclear Medicine, Wuxi, 214063 Jiangsu People’s Republic of China; 3 Jiangsu Key Laboratory of Pathogen Biology, Department of Pathogen Biology and Immunology, Nanjing Medical University, Nanjing, 211166 Jiangsu People’s Republic of China; 4 Department of Emergency Center, The First Affiliated Hospital of Nanjing Medical University, Nanjing, 210029 Jiangsu People’s Republic of China; 5 Department of Pathology, The Affiliated Hospital of Jiangsu University, Zhenjiang, 212000 Jiangsu People’s Republic of China; 6 Department of Central Laboratory, The Affiliated Hospital of Jiangsu University, Zhenjiang, 212000 Jiangsu People’s Republic of China; 7 Department of Respiratory Diseases, The Affiliated Huai’an Hospital of Xuzhou Medical University, Huai’an, 223002 Jiangsu People’s Republic of China

**Keywords:** PELI1, PI3K/AKT, miR-30c-5p, hUCMSC-EVs, Papillary thyroid carcinoma

## Abstract

**Background:**

The aberrant expression of E3 ubiquitin ligase Pellino-1 (PELI1) contributes to several human cancer development and progression. However, its expression patterns and functional importance in papillary thyroid cancer (PTC) remains unknown.

**Methods:**

*PELI1* expression profiles in PTC tissues were obtained and analyzed through the starBase v3.0 analysis. Real-time PCR, Immunohistochemical assays (IHC) and Western blot were used to investigate the mRNA and protein levels of PELI1 in PTC. The effects of PELI1 on PTC cell progression were evaluated through CCK-8, colony formation, Transwell, and Wound healing assay in vitro, and a PTC xenograft mouse model in vivo. The downstream target signal of PELI1 in PTC was analyzed by using Kyoto encyclopedia of genes and genomes (KEGG), and bioinformatics tools were used to identify potential miRNAs targeting PELI1. Human umbilical cord mesenchymal stem cells were modified by miR-30c-5p and the miR-30c-5p containing extracellular vesicles were collected (miR-30c-5p-EVs) by ultra-high-speed centrifugation method. Then, the effects of miR-30c-5p-EVs on PELI1 expression and PTC progression were evaluated both in vitro and in vivo.

**Results:**

Both mRNA and protein expression of PELI1 were widely increased in PTC tissues, and overexpression of *PELI1* was positively correlated with bigger tumor size and lymph node metastases. PELI1 promoted PTC cell proliferation and migration in vitro. While, PELI1 silencing significantly suppressed PTC growth in vivo accompanied with reduced expression of Ki-67 and matrix metallopeptidase 2 (MMP-2). Mechanistically, PI3K-AKT pathway was identified as the downstream target of PELI1, and mediated the functional influence of PELI1 in PTC cells. Moreover, we found that the expression of miR-30c-5p was inversely correlated with PELI1 in PTC samples and further confirmed that miR-30c-5p was a tumor-suppressive miRNA that directly targeted PELI1 to inhibit PTC cell proliferation and migration. Furthermore, we showed that miR-30c-5p-EVs could effectively downregulate PELI1 expression and suppress the PTC cell growth in vitro and in vivo.

**Conclusion:**

This study not only supported the first evidence that miR-30c-5p loss-induced PELI1 accumulation facilitated cell proliferation and migration by activating the PI3K-AKT pathway in PTC but also provided novel insights into PTC therapy based on miR-carrying-hUCMSC-EVs.

**Supplementary Information:**

The online version contains supplementary material available at 10.1186/s12967-021-03226-1.

## Background

The incidence of thyroid cancer (THCA) has ascended steadily worldwide over the past decades, and this disease is projected to become the fourth leading type of cancer [1]. Papillary thyroid cancer (PTC) is the most common subtype, accounting for approximately 85% of all THCA. Although patients with PTC may experience long-term and disease-specific survival after surgical treatment, poor prognosis often occurs and that is closely associated with tumor regional recurrence or metastasis [2]. Therefore, better understanding the molecular mechanisms in PTC and identifying novel targets are still eagerly needed.

E3 ubiquitin ligases are a class of critical enzymes that can catalyse the transfer of ubiquitin to the substrate, and their genetic alteration, abnormal expression, or dysfunction account for the occurrence and progression of human cancers [3]. Therefore, they are emerging as attractive therapeutic targets for cancer therapy [4]. Pellino-1 (PELI1), a novel cancer-related E3 ubiquitin ligase, has been found to be upregulated and correlated with poor clinical prognosis in a variety of cancers, such as large B cell lymphomas [5, 6], lung cancer [7, 8] and breast cancer [9]. In addition, PELI1 could regulate the metabolic actions of mTORC1 to suppress antitumor T cell responses in melanoma [10]. These reports identified PELI1 was an oncogene. However, a recent study reported that PELI1 could promote radiotherapy sensitivity by inhibiting noncanonical NF-κB in esophageal squamous cancer [11], suggesting that PELI1 was a tumor suppressor. Nevertheless, the expression patterns and functional importance of PELI1 in PTC are not known.

MicroRNAs (miRNAs), which are small non-coding RNAs, modulate the expression of cognate target genes by interacting with their mRNA 3′-untranslated region (3′-UTR), resulting in mRNA degradation or translational inhibition [12]. Obviously, miRNAs are gene’s fine-tuner [13, 14]. Accumulating study reveal that miRNAs can act as novel types of cancer ontogenesis or suppressors in tumor carcinogenesis [15]. In particular, miRNAs are closely related to PTC development and progression [16]. Therefore, whether Peli1 expression is regulated by certain miRNAs as a posttranscriptional regulation mechanism in PTC is worth exploring.

Here, we found that PELI1 was widely unregulated in PTC samples and cells. PELI1 promoted PTC cell proliferation and migration, which was associated with the activation of PI3K-AKT signaling pathway. Notably, PELI1 was inversely regulated by miR-30c-5p and was a direct functional target of miR-30c-5p in PTC. Moreover, miR-30c-5p could be incorporated by human umbilical cord mesenchymal stem cells (hUCMSC) and released in extracellular vesicles (EVs), which could inhibit PELI1 expression and PTC growth in vitro and in vivo. Our findings not only provided that miR-30c-5p/PELI1/PI3K-AKT axis might function as a key pathway regulating PTC progression, but also showed a novel translational therapeutic approach for PELI1 inhibition and PTC treatment based on miR-30c-5p-carrying-hUCMSC-EVs.

## Materials and methods

### Human samples

PTC tissues and human umbilical cord samples were obtained from informed and consenting PTC patients and mothers respectively at the Affiliated Hospital of Jiangsu University (Zhenjiang, China). This study was approved by the Ethics Committee of the Affiliated Hospital of Jiangsu University.

### Cell culture

W3, TPC-1 and Nthy-ori 3–1 were purchased from Cell Bank of Chinese Academy of Sciences and were cultured in DMEM medium (Gibco, Carlsbad, CA) containing 10% FBS (Gibco) and 1% pen/strep (Gibco) in a humidified atmosphere at 37 °C with 5% CO_2_. HUCMSCs were isolated from fresh umbilical cord samples as previously described [17, 18] and maintained in stem cell culture medium (Cyagen, Guangzhou, China).

### Lentivirus transduction and oligonucleotide transfection

Lentiviral particles for PELI1 overexpression (LV-PELI1), inhibition constructs (sh-PELI1), and their relative control (LV-NC; sh-NC, respectively) were packaged and purchased from GeneChem (Shanghai, China). PTC cells were infected with recombinant lentivirus transducing units plus 5 μg /ml polybrene (Sigma, Natick, MA) according to the manufacturer's manual. *PELI1* shRNA sequences: 5′-GCCAAATGGAAGACATCAGAT-3′; scrambled shRNA: 5′- TTCTCCGAACGTGTCACGT-3′.

The synthetic miR-30c-5p mimics, mimic control, miR-30c-5p inhibitor and inhibitor control were purchased from GenePharma (Shanghai, China). The plasmids of pcDNA-Peli1 and the empty vector were kindly gifted from Professor Yichuan Xiao (Chinese Academy of Sciences, Shanghai, China). PTC cells were transfected with 50 nM miR-30c-5p mimic or 100 nM miR-30c-5p inhibitor or miR-30c-5p mimic plus 1 μg Peli1 plasmid using Lipofectamine 2000 (Invitrogen, Carlsbad, CA) according to the manufacturer’s protocol.

### HUCMSC-EVs engineered by miR-30c-5p (miR-30c-5p-EVs)

HUCMSCs were transfected with 50 nM miR-30c-5p mimic or mimic NC using Lipofectamine 2000 (Invitrogen), followed by culturing with conditioned medium [19]. MiR-30c-5p-EVs or NC-EVs were isolated from the conditioned medium by using ultra-high-speed centrifugation (Beckman Coulter Optima L-100 XP ultracentrifuge, Miami, FL) as our previously described [17, 18].

Morphology of the miR-30c-5p-EVs was observed using transmission electron microscopy (JEM-1200EX; JEOL Ltd., Tokyo, Japan). The particle number and size distribution of the EVs were determined by using ZetaView PMX 110 (Particle Metrix, Meerbusch, Germany) according to the manufacturer’s manual.

### Cell treatment

PELI1-overexpressing PTC cells (LV-PELI1) and their control cells (LV-NC) were seeded in 6-well plates. After culturing overnight, the LV-PELI1 cells were treated with LY294002 (20 μM; MedChem Express, Monmouth Junction, NJ) dissolved in DMSO or DMSO alone. After incubation of 12 h, the cells were harvested for protein extraction.

PTC cells were seeded in six-well plates (10% EVs-free FBS complete DMEM medium) and treated with PBS, hUCMSC-EVs (10^4^ particles/cell), NC-EVs (10^4^ particles/cell) or miR-30c-5p-EVs (10^4^ particles/cell) daily for three days as previous described [20].

### Cell proliferation assay

Cell viability was determined using CCK-8 assays (KeyGEN BioTECH, Nanjing, China) following the manufacturer’s protocol. Briefly, PTC cells were seeded in 96-well plates (2 × 10^3^/well). After the indicated hours (24, 48, 72 and 96) of incubation, CCK-8 solution (10 μL) was added to each well, followed by incubated for another 2 h. The absorbance was measured at 450 nm using a microplate reader (Synergy HT, BioTek, Biotek Winooski, VT).

For colony formation assay, W3 and TPC-1 cells transfected with lentivirus, oligonucleotide, or treated by EVs were seeded in 6-well plates (1000/well) and cultured in medium containing 10% FBS. After 10 days, the cells were fixed with methanol and stained with 0.4% crystal violet solution, finally photographed. Colonies containing more than 50 cells were counted.

### Cell migration assay

PTC cells were plated into the upper Transwell chamber (W3: 5 × 10^4^; TPC-1: 3 × 10^4^ in 200 μl serum-free medium). The lower chamber was filled with 600 μl medium supplemented with 10% FBS. After 12 h of incubation, cells on the upper membrane surface were removed and the membranes were fixed with methanol, stained with 0.4% crystal violet solution, imaged using a Nikon microscope.

For wound healing assay, the monolayer cells were scratched by a 200-μl pipette tip. Separated cells were washed out using PBS, and images of the same fields at 48 h after the scratch were recorded under a microscope.

### Mouse model study

Four-week-old female BALB/c nude mice were purchased from the Comparative Medicine Centre of Yangzhou University (Yangzhou, China).

To evaluate the effect of PELI1 on PTC growth, the W3 cells stably transfected with sh-PELI1 or control sh-NC (2 × 10^6^ cells) were injected subcutaneously into the right or left of the mice flank respectively.

To investigate the effect of miR-30c-5p-EVs on PTC growth, nude mice were inoculated subcutaneously on right flanks with W3 cells (2 × 10^6^ cells). After 7 days of tumor growth, PBS (20 μL), hUCMSC-EVs, NC-EVs, or miR-30c-5p-EVs (all kind of EVs: 2 × 10^10^ in a volume of 20 μL of PBS) were administered via intra-tumor injection weekly described by Bruno et al. [19]. On day 28, tumors were removed for examination. Tumor volume in mm^3^ was calculated from the length (L) and width (w) axis of the tumors using the following formula: V = L × W^2^/2.

### RNA isolation and quantitative real-time PCR

Total RNA was isolated using Trizol reagent (Invitrogen) or mirVana RNA isolation kit (Ambion, Austin, TX) according to the manufacturer’s protocol. Real-time PCR was performed with All-in-one™ qPCR Mix (Genecopoeia) in a QuantStudio 5 Real-Time system (Thermo Fisher Scientific, Waltham, MA). All of the primers for real-time PCR, including PELI1 (HQP0504204), β-actin (HQP016381), Has-miR-30c-5p (HmiRQP0396) and U48 (HmiRQP9021) were purchased from Genecopoeia (Guangzhou, China). The relative expression of *PELI1* and miR-30c-5p was normalized to β-actin and U48 respectively, and evaluated by the 2^−ΔΔCt^ method, based on our previous description [21, 22].

### Immunohistochemical (IHC) assay

IHC analyses of tissues were conducted as described in previous study [17, 23]. Briefly, PTC tissue sections were incubated with an antibody to PELI1 (12,053–1-AP, diluted 1: 200; Proteintech, Rosemont, IL); mice tumor sections were incubated with an antibody to Ki-67 (ab16667, diluted 1: 200; Abcam, Cambridge, MA) or MMP2 (10,373–2-ap, diluted 1: 200; Proteintech) overnight at 4 °C, followed incubating by HPR-conjugated secondary antibody. Diaminobenzidine was used as the substrate. The nuclei were counterstained with hematoxylin. All pictures were captured using a Nikon microscope. Integrated optical density of Ki-67 and MMP2 in mice tumor sections were measured by using Image-Pro Plus software (Version X; Adobe, San Jose, CA).

### Western blotting

The proteins were extracted using RIPA buffer (Cell Signaling Technology Inc., Danvers, MA) and quantified using a BCA Protein Kit (Beyotime). Western blot analysis was carried out as described previously [21]. The antibodies against PELI1 (sc-271065) was purchased from Santa cruz biotechnology (Santa Cruz, CA); Phospho-AKT (ab81283), AKT (ab179463), Ki-67 (ab16667), TSG101 (ab133586), HSP70 (ab181606) and HRP-linked anti-rabbit/mouse IgG (ab97051/ ab6728) were purchased from Abcam; MMP2 (10,373–2-ap) and GAPDH (60,004–1-lg) were purchased from Proteintech. The intensity of protein bands was quantitated using Image J (National Institutes of Health, Bethesda, MD), and data were normalized against that of the corresponding GAPDH bands.

### Luciferase reporter assay

PTC cells were co-transfected with 500 ng pmiR-RB-report-h-*PELI1*-3′UTR (wild type and mutant type; GeneCopoeia) and 50 nM miR-30c-5p miRNA (mimics and mimic NC; GenePharma) using Lipofectamine 2000 (Invitrogen). After 48 h, cells were collected and their luciferase activity was measured using the Luc-Pair™ Duo-Luciferase HS Assay Kit (GeneCopoeia). The results are expressed as the relative firefly luciferase activity, which is obtained after normalization to Renilla luciferase activity.

### Statistical analysis

The statistical analyses were performed with GraphPad Prism (Version 5.0; La Jolla, CA). Data are expressed as mean ± SD. The groups were compared using the Student’s t-test or one-way analysis of variance (Tukey Kramer post hoc tests). *P* < 0.05 was considered statistically significant.

## Results

### PELI1 is upregulated in human PTC samples and cells

**Fig. 1:**
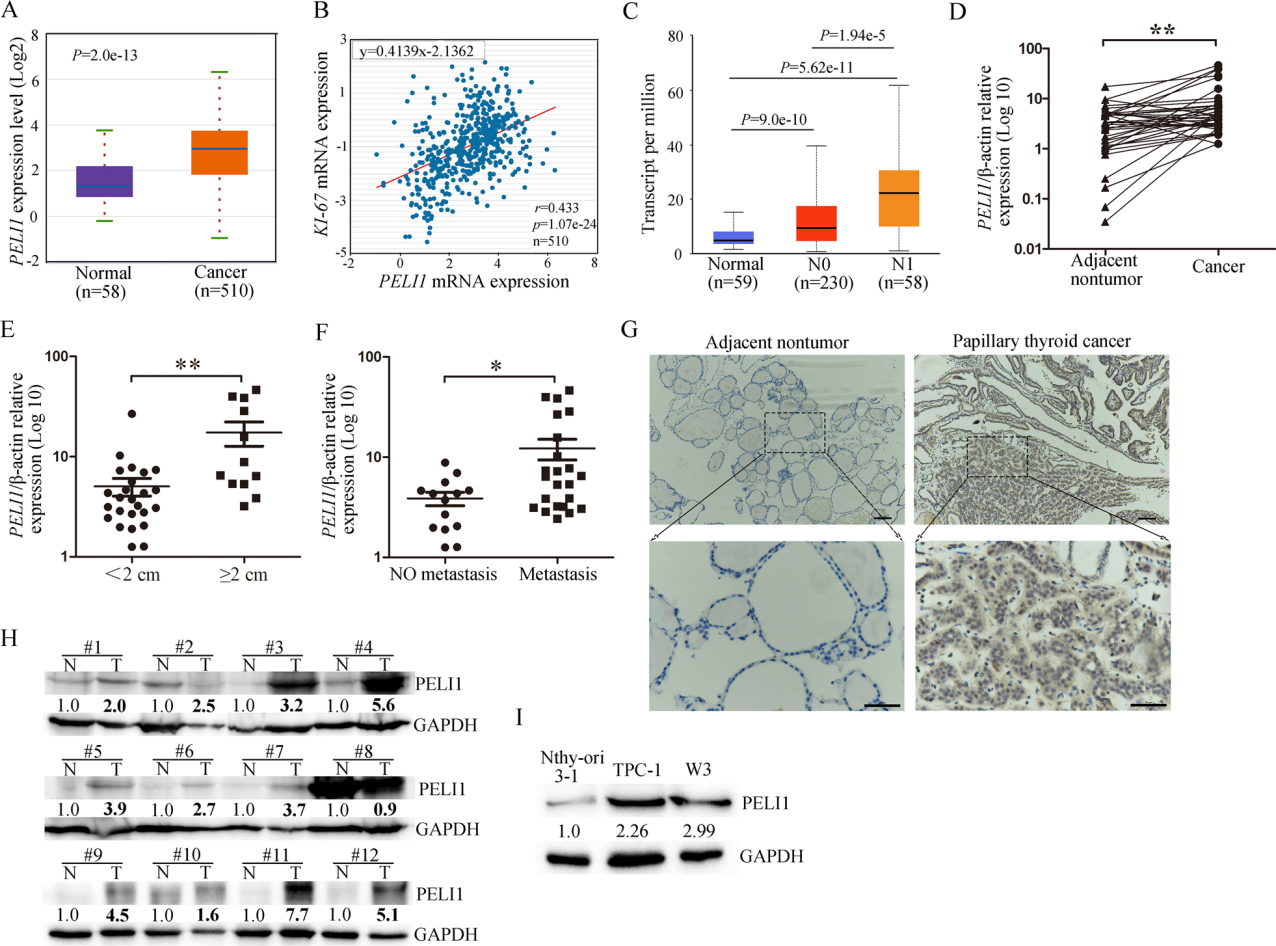
PELI1 is highly expressed in PTC tissues and cell lines. **A** StarBase v3.0 dataset (http://starbase.sysu.edu.cn/) showed that *PELI1* was significantly upregulated in THCA tissues compared with normal tissues. **B** Correlation analysis of *PELI1* and *Ki-67* in THCA using starBase v3.0. **C** MRNA expression level of *PELI1* in normal tissues, thyroid carcinoma tissues with no regional lymph node metastasis (N0) and thyroid carcinoma tissues with metastases in 1 to 3 axillary lymph nodes (N1) using the UALCAN dataset (http://ualcan.path.uab.edu/index.html). **D** Relative *PELI1* expression levels in adjacent normal tissues and PTC tumor tissues were determined by Real-time PCR (n = 37). **E**, **F** Real-time PCR analysis showing the pattern of *PELI1* expression in PTC tumor diameter ≥ 2 cm compared with tumor diameter < 2 cm (**E**), and metastatic PTC compared with non-metastatic PTC (**F**). **G** A representative images of IHC staining for PELI1 in human PTC tissues and adjacent non-tumor tissues (n = 10). The scale bars represent 100 µm (upper panel) and 50 µm (lower panel). **H** Western blotting analyses performed using human PTC tissues (T) and adjacent non-tumor tissues (N). Band intensities were normalized against the corresponding GAPDH and the numbers are presented as fold increase over the N group, respectively. **I** Nthy-ori 3–1, TPC-1 and W3 were analyzed for PELI1 expression using western blotting and a representative’s result was showed from two independent experiments. *P < 0.05, **P < 0.01

First, mRNA expression of PELI1 in PTC was analyzed using Starbase (http://starbase.sysu.edu.cn/index.php). We found that the *PELI1* expression was significantly increased in THCA specimens compared with normal tissues (Fig. 1A). Pearson correlation test showed that *PELI1* was positively correlated with *Ki-67* (a frequently used proliferation marker) in PTC samples (Fig. 1B). In addition, the analysis of data from UALCAN (http://ualcan.path.uab.edu/index.html) showed that *PELI1* was expressed at higher levels in THCA tissues from patients with lymph node metastases (Fig. 1C).

Then, the expression of *PELI1* in PTC and corresponding adjacent tissues was determined by real-time PCR. *PELI1* expression was significantly unregulated in PTC samples than that in adjacent tissues (Fig. 1D). Moreover, patients with bigger tumor size (≥ 2 cm) exhibited higher *PELI1* level than patients with small tumor size (< 2 cm, Fig. 1E). In addition, *PELI1* expression increased in metastatic PTC tissues compared with the primary PTC tissues (Fig. 1F). Consistent with the mRNA levels, the PELI1 protein levels were higher in PTC tissues than in adjacent non-tumor tissues, and PELI1 was predominantly localized in the nucleus and cytoplasm of PTC cells based on IHC (Fig. 1G). The protein expression of PELI1 in PTC tissues was also investigated using Western blot, and higher expression of PELI1 was observed in PTC tissues than in matched adjacent normal tissues (Fig. 1H). Furthermore, the protein expression of PELI1 was expressed at higher levels in PTC cell lines (W3 and TPC-1) than in the normal human thyroid cell line Nthy ori-3–1 (Fig. 1I). Collectively, these findings indicated that PELI1 was upregulated in PTC and might play an oncogenic role in the progression of PTC.

### PELI1 promotes PTC cell proliferation and migration in vitro and knockdown of PELI1 suppresses PTC tumor growth in vivo

**Fig. 2:**
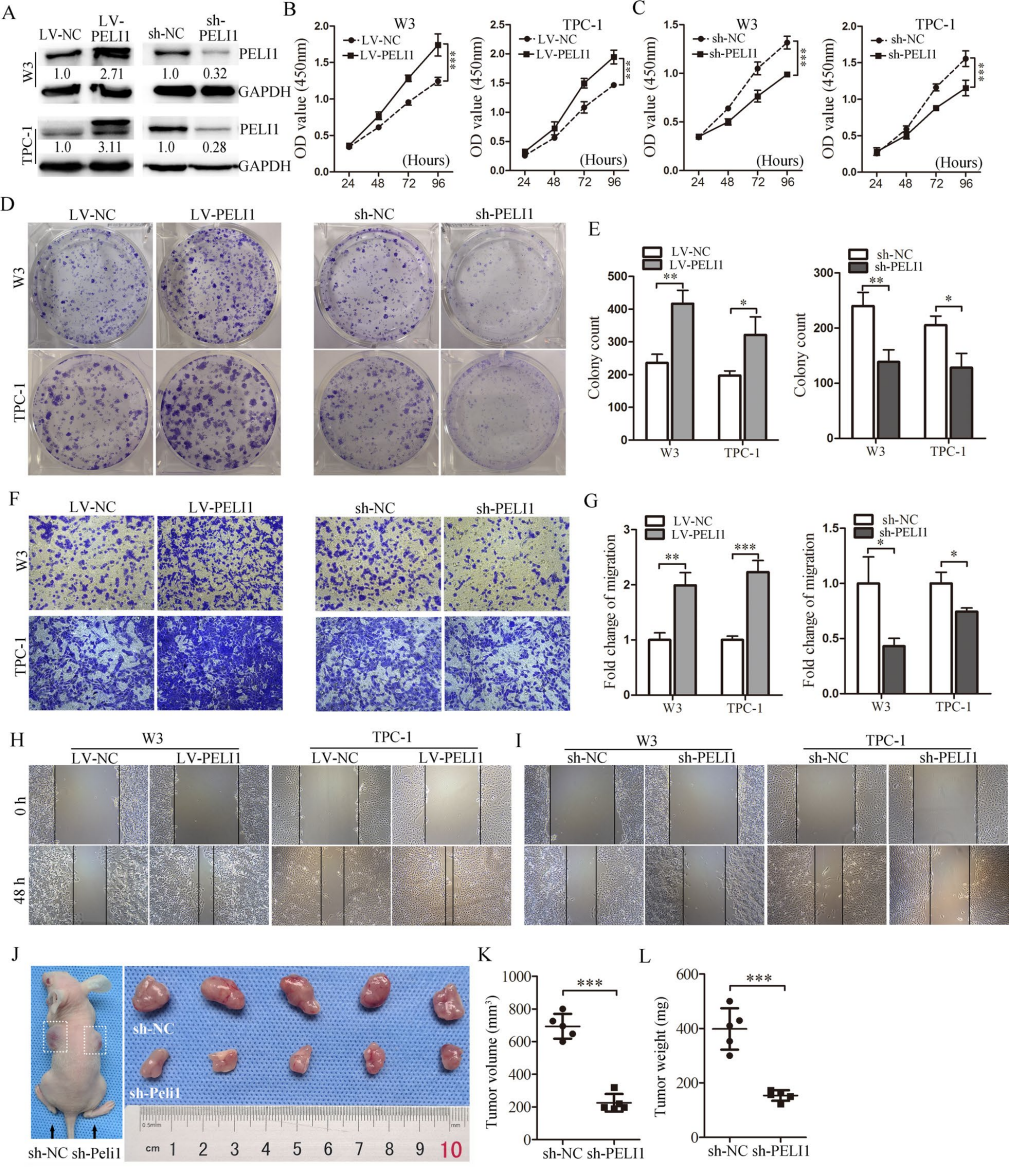
PELI1 overexpression enhances the cellular proliferation, migration and oncogenic transformation in PTC cells. **A** Western blottings showing the PELI1 levels in lentiviral infected W3 and PTC-1 cells. The results are representative of three independent experiments. **B**, **C** CCK8 assay was performed to estimate the cell proliferation of W3 and TPC-1 cells with PELI1 overexpression (**B**) or knock-down (**C**) at 24, 48, 72 and 96 h (n = 5). **D**, **E** W3 and TPC-1 cells with PELI1 overexpression or PELI1 depletion were subjected to colony-forming assay, the representative micrographs of crystal violet-stained cell colonies were displayed (**D**), and the quantification of colony formation assays was shown (n = 3) (**E**). **F**, **G** Transwell assay was performed to estimate the cell migration of W3 and TPC-1 cells with PELI1 overexpression or knock-down, the representative images (× 200) of Transwell assays were displayed (**F**), and the quantification of Transwell assays was shown (n = 3) (**G**). **H**, **I** Representative images (× 100) of scratch wound healing assays of W3 and TPC-1 cells stably expressing PELI1 or knock-down (n = 3). **J**–**L** Xenograft model in nude mice and the tumors were showed, and tumor volume (**K**) and tumor weight (**L**) were analysis. *P < 0.05, **P < 0.01, ***P < 0.001

To evaluate the functional significance of PELI1 in PTC tumorigenesis, we overexpressed or knocked down the protein of PELI1 in TPC-1 and W3 cells by transfection with lentiviral particles for PELI1 overexpression (LV-PELI1) or inhibition (sh-PELI1), respectively (Fig. 2A). Cell counting kit-8 (CCK-8) assay results revealed that PELI1 overexpression significantly increased PTC cell proliferation (Fig. 2B), whereas the knockdown of PELI1 markedly reduced cell proliferation (Fig. 2C). A similar effect was observed in colony-forming assay, where the colony numbers of PTC cells were markedly increased upon PELI1 overexpression and decreased upon PELI1 knockdown (Fig. 2D and E). To validate the function of PELI1 on the migration of PTC cells, we carried out Transwell migration assay, in which the overexpression of PELI1 enhanced cell migration, whereas the depletion of PELI1 suppressed the number of migrated PTC cells (Fig. 2F and G). Similarly, the results of the scratch wound assay revealed that PELI1 overexpression increased the migration of TPC-1 and W3 cells (Fig. 2H). However silencing PELI1 decreased the migration ability of both TPC-1 and W3 cells (Fig. 2I).

To determine whether PELI1 may be served as a potential target for PTC therapeutics in vivo, PTC cells (W3) stably transfected with sh-PELI1 or control were subcutaneously injected into nude mice. All the nude mice developed xenograft tumors at the injection sites (Fig. 2J). As shown in Fig. 2K and L, the average volume and weight of tumors in the sh-PELI1 group were significantly lower than those in the sh-NC group. Taken together, these findings suggested that PELI1 functions as an oncogene to promote PTC cell progression.

### PELI1 promotes PTC cell proliferation and migration by activating the PI3K-AKT pathway

**Fig. 3:**
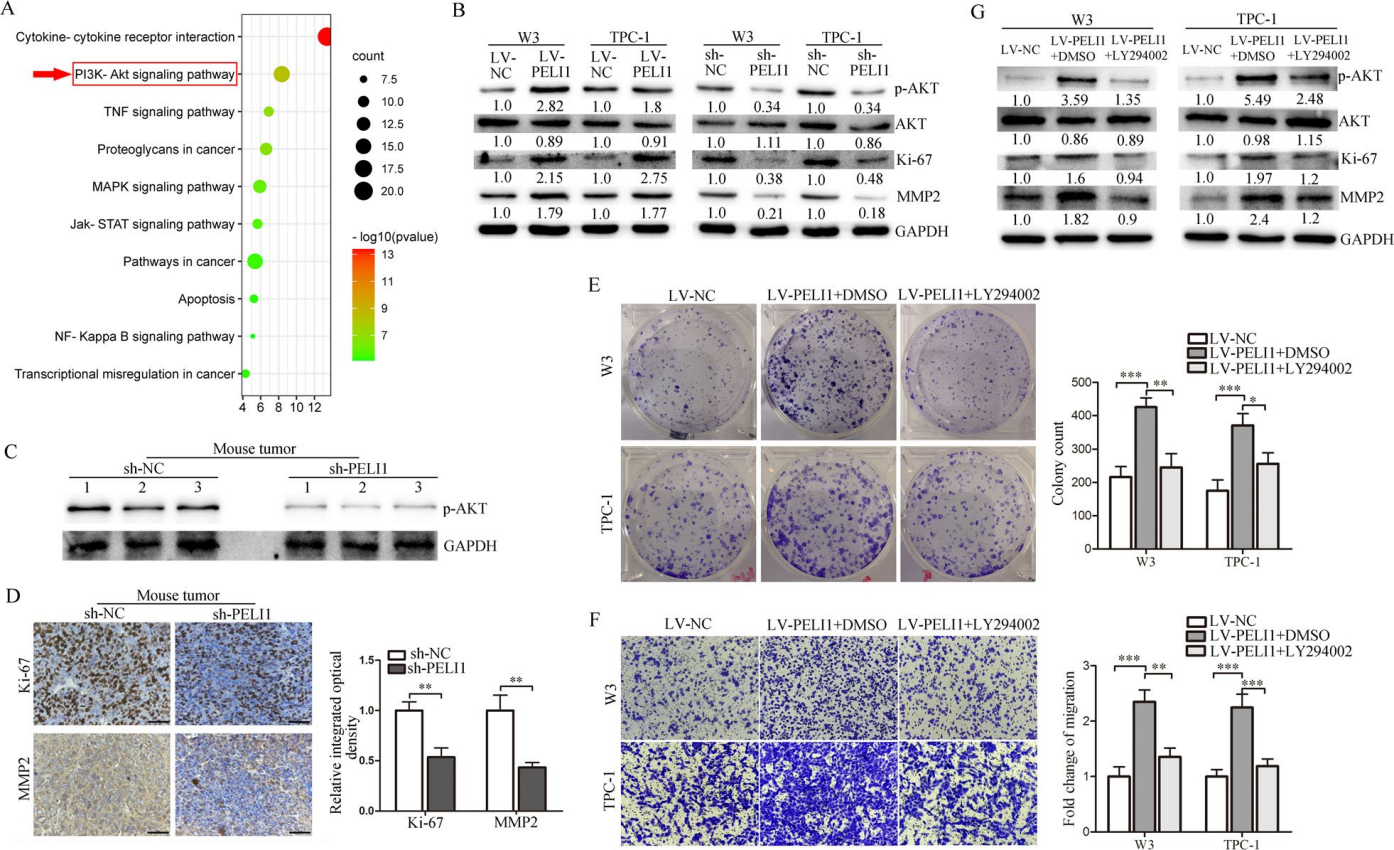
PELI1 activates the PI3K-AKT signaling pathways. **A** KEGG enrichment analysis showed that genes co-expressed with *PELI1* are enriched in varied of pathway. **B** The protein levels of p-AKT, AKT, Ki-67 and MMP2 were assessed by western blotting in PELI1-overexpression or Peli1-silencing PTC cells. **C** Western blotting analysis showing the levels of PELI1 and p-AKT in the mouse tumor samples. **D** Representative IHC staining pictures of Ki-67 and MMP2 from mouse tumor sections (left panel), and the quantification of Ki-67 and MMP2 expression (right panel) (n = 3). The scale bars represent 50 µm. **E** Colony formation assay of PELI1-overexpression PTC cells after PI3K/AKT inhibition (LY294002; left panel), and the quantification was shown (right panel; n = 3). **F** Transwell assay was performed to estimate the cell migration of PELI1-overexpressing PTC cells after PI3K/AKT inhibition, the representative images (× 200) of Transwell assays were displayed (left panel), and the quantification was shown (right panel; n = 3). **G** Inhibition efficiency of PI3K/AKT and protein levels of Ki-67 and MMP2 in PELI1-overexpressing PTC cells after LY294002 treatment were determined by western blotting. The numbers are presented as fold increase over the LV-NC group. *P < 0.05, **P < 0.01, ***P < 0.001

E3 ubiquitin ligases can influence the cancer progression by regulating signal pathways [24, 25]. To explore how PELI1 exerts its oncogenic activity in PTC, a list of 270 genes that have high Pearson correlation coefficient value (> 0.5) with *PELI1* (called co-expressed genes) were selected from TCGA THCA databases (Additional file 1). Then, we performed KEGG analysis on these genes using KOBAS-I (http://kobas.cbi.pku.edu.cn/) to identify the possible downstream signaling pathways of PELI1. The significantly (*P* < 0.001) enriched pathways were related to cytokine-cytokine receptor interaction, PI3K-AKT, TNF signaling, proteoglycans in cancer, MAPK, JAK-STAT, pathways in cancer, apoptosis, NF-κB and transcriptional misregulation in cancers (Fig. 3A). Among these pathways, PI3K-AKT (indicated by the arrows) was selected for further analysis (Fig. 3A), because a relatively large number of co-expressed genes (such as *BCL2L1*, *YWHAZ*, *ITGB4*, *ITGA9*, *CDKN1A*, and so on; Additional file 2) were associated with this pathway and PI3K-AKT signaling plays a crucial role in thyroid carcinogenesis and metastasis [26, 27]. Compared to the control cells, the protein level of AKT phosphorylation (p-AKT) upregulated in cells overexpressing PELI1, while decreased in PELI1-silenced cells, but the total AKT protein had no change. In addition, the downstream effectors of PI3K-AKT signal, including Ki-67 and MMP2 total proteins [28], which are related to cell proliferation and migration, were enhanced in cells with PELI1 overexpression but suppressed in cells with PELI1 knockdown (Fig. 3B). In addition, tumors tissues derived from the sh-PELI1 mice (same sample with Fig. 2J) showed less expression of p-AKT than those in the sh-NC group (Fig. 3C), and the IHC staining results revealed that both Ki-67 and MMP2 were significantly downregulated by the knockdown of PELI1 (Fig. 3D).

Then, we inhibited PI3K/AKT pathway in PELI1-overexpressing PTC cells by using LY294002 (PI3K inhibitor) [8]. Interestingly, the enhanced cell proliferation and migration in PTC cells caused by PELI1 overexpression was dramatically suppressed by LY294002 (Fig. 3E and F). Additionally, protein levels of Ki-67 and MMP-2 in PELI1-overexpression TPC cells was decreased (Fig. 3G). Therefore, PI3K/AKT signaling pathway was critical for PELI1-mediated oncogenic function in PTC.

### MiR-30c-5p loss induces PELI1 upregulation in PTC

**Fig. 4:**
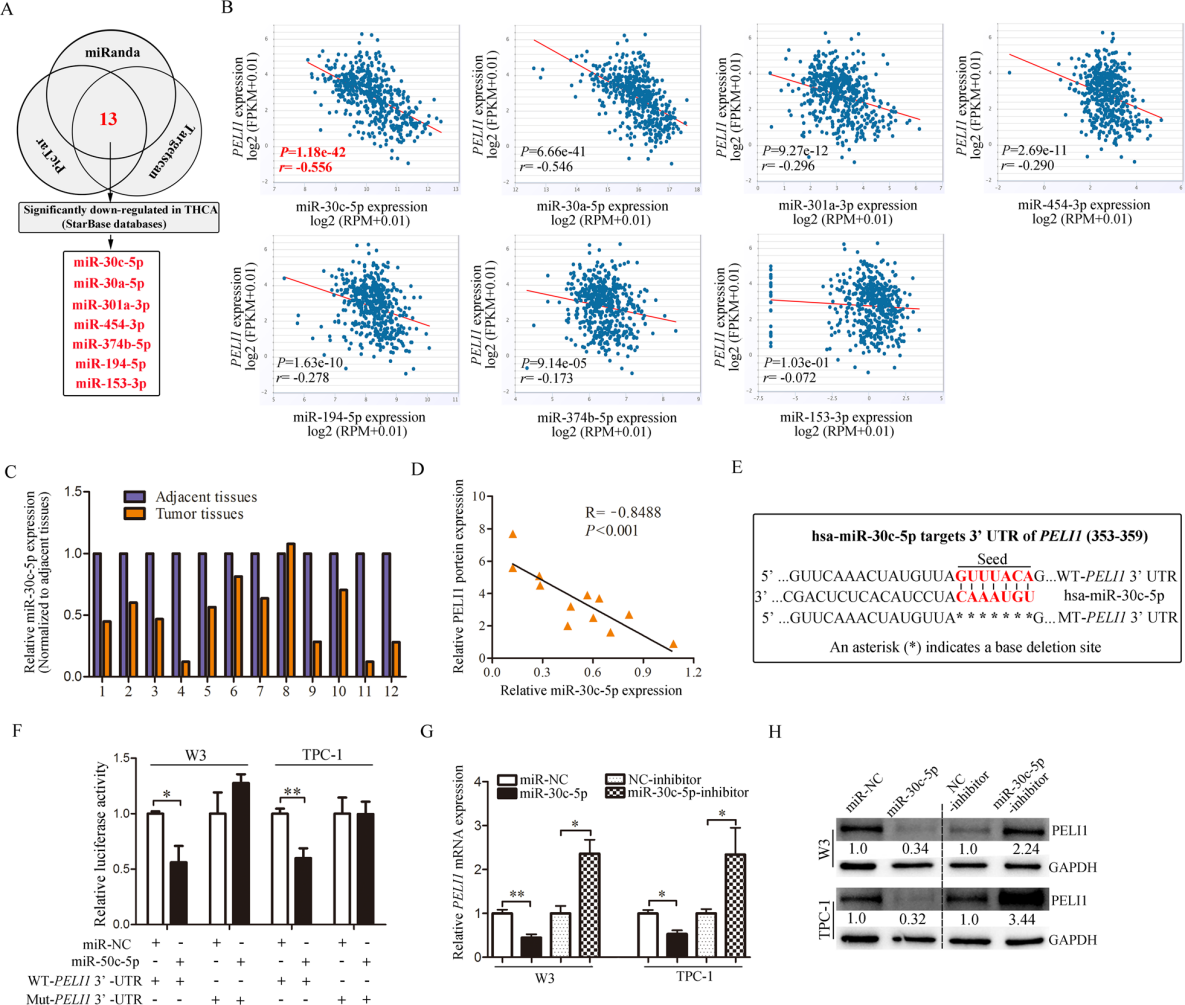
PELI1 is a direct target of miR-30c-5p in PTC cells. **A** Overlap of miRNAs targeting *PELI1* in the PicTar, miRanda and Targetscan, and miRNAs significantly downregulated in THCA form starBase datasets. **B** Correlation analysis of miR-30c-5p, miR-30a-5p, miR-301a-3p, miR-454-3p, miR-194-5p, miR-374b-5p, miR-153-3p, and *PELI1* in THCA using starBase. **C** MiR-30c-5p was detected in 12 pairs of PTC tissues by real-time PCR. **D** Relationship between miR-30c-5p and PELI1 protein in 12 pairs of PTC tissues was measured. **E** Predicted miR-30c-5p target sequences (red) in *PELI1* 3′-UTR. Seven nucleotides (*) were mutated to prevent binding to miR-30c-5p. **F** Relative luciferase activity of reporter plasmids carrying wild-type or mutant *PELI1* 3′-UTR in PTC cells co-transfected with miR-NC or miR-30c-5p mimics (n = 4). 44% and 41% are the mean reduction values of four duplicate from one experiment and the corresponding *P*-value is 0.0147 and 0.004 respectively. **G** Real-time PCR analysis of the expression of *PELI1* in PTC cells at 24 h post transfection of the miR-30c-5p mimics or miR-30c-5p inhibitor compared to their negative controls (n = 3). **H** Level of PELI1 in PTC cells after miR-30c-5p mimics or miR-30c-5p inhibitor transfected was analyzed by western blotting. *P < 0.05, **P < 0.01, ***P < 0.001

To elucidate why PELI1 increased in PTC, firstly, we used miRNA target prediction software TargetScan, PicTar and miRanda to identify the potential miRNAs targeting *PELI1*. The intersection of these three databases screened 13 highly reliable miRNAs. Then, we used StarBase databases to check whether these 13 miRNAs were significantly down-regulated in THCA. After analysis, it was found that miR-30c-5p, miR-30a-5p, miR-301a-3p, miR-454-3p, miR-374b-5p, miR-194-5p and miR-153-3p were significantly down-regulated in THCA, and the remaining 6 miRNAs were upregulated or had no significant difference (Fig. 4A and Additional file 3). Next, we performed StarBase Pan-Cancer Analysis to validate the correlation between *PELI1* and these 7 significantly down-regulated miRNAs in THCA. Intriguingly, among these 7 miRNAs, miR-30c-5p was mostly significantly inversely correlated with *PELI1* mRNA expression (Fig. 4B). Therefore, miR-30c-5p was regarded as the focus of attention. Then, 12 collected paired PTC tissues (same sample with Fig. 1G) were used to investigate the association between miR-30c-5p expression and the protein level of PELI1. We found that compared with the adjacent normal tissues, a total of 11 PTC samples had lower miR-30c-5p expression, and one had higher expression (Fig. 4C), and as expected, miR-30c-5p levels were significantly inversely correlated with PELI1 protein levels (Fig. 4D).

To verify whether miR-30c-5p directly binds the 3′-UTR region of *PELI1*, we conducted chimeric constructs, which harbor luciferase wild-type 3′-UTR sequence (WT-*PELI1*-3′-UTR) or mutant 3′-UTR sequence (MT-*PELI1*-3′-UTR) (Fig. 4E), and then performed dual-luciferase reporter assay in PTC cells. As shown in Fig. 4F, miR-30c-5p mimics significantly repressed the luciferase activity of the reporter gene within the wild-type construct (44% and 41% decreases in W3 and TPC-1 cells, respectively) but not the mutant *PELI1* 3′-UTR construct. We then transfected mimics or inhibitors of miR-30c-5p in PTC cells and examined the effect of miR-30c-5p on PELI1 expression. Results of real-time PCR (Fig. 4G) and western blot (Fig. 4H) showed that miR-30c-5p mimics reduced the mRNA and protein levels of PELI1, and the inhibitors of miR-30c-5p increased the mRNA and protein levels of PELI1 in W3 and TPC-1 cells. Together, these findings suggested that miR-30c-5p downregulation induced PELI1 upregulation in PTC.

### Overexpression of miR-30c-5p can inhibit PTC cell proliferation and migration

**Fig. 5:**
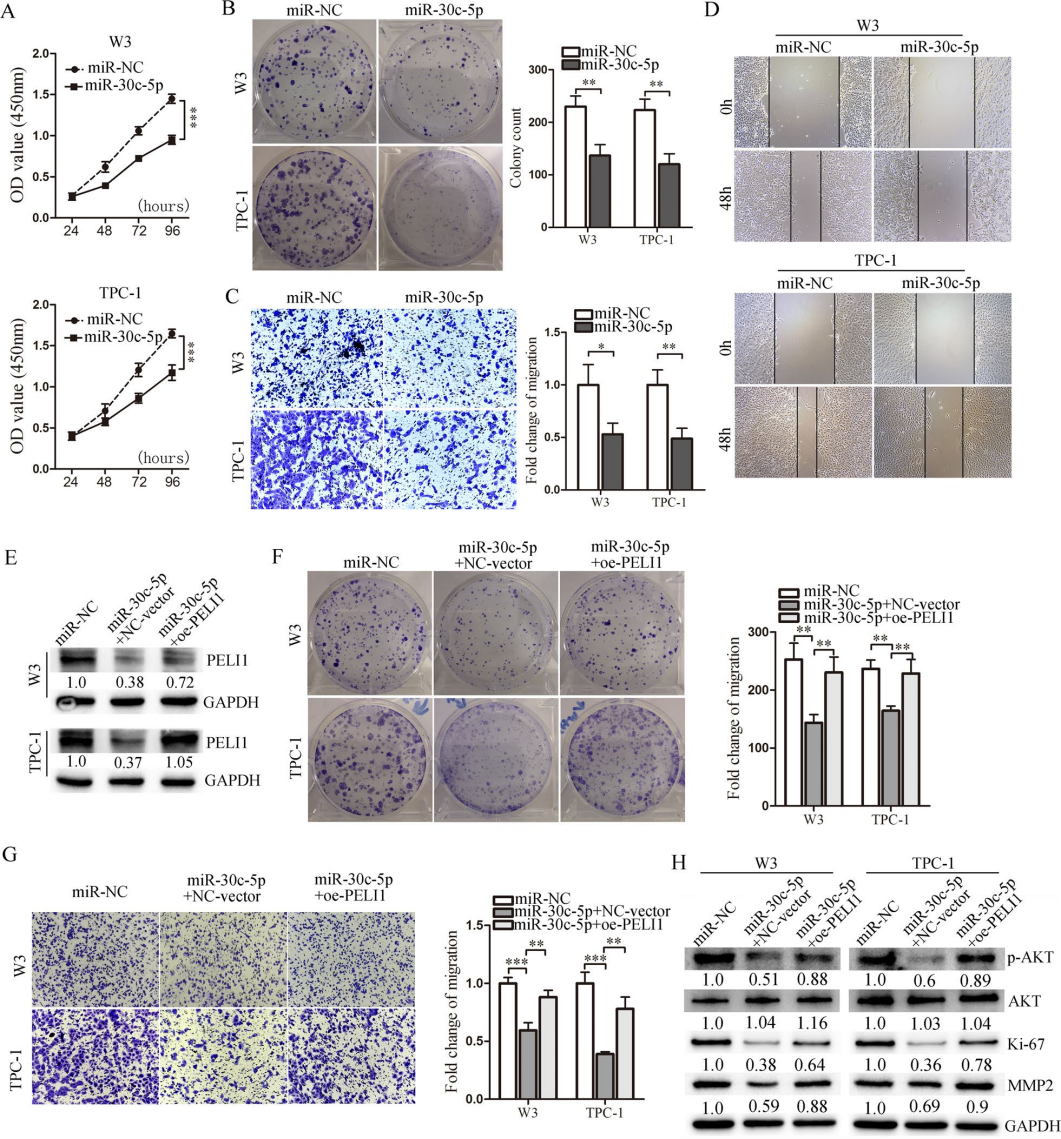
MiR-30c-5p inhibits PTC cell proliferation and migration by downregulating PELI1. **A** The cell proliferation of PTC cells with miR-30c-5p mimic or its control transfection was detected by CCK8 assay (n = 5). **B** Colony formation assays of PTC cells transfected with miR-NC or miR-30c-5p, and the quantification of colony formation was shown (n = 3). **C** Representative images of Transwell assays of PTC cells transfected with miR-30c-5p, and the quantification was shown (n = 3). **D** Representative images of scratch wound healing assays of PTC cells transfected with miR-30c-5p. **E** Transfection efficiency of increased PELI1 overexpression (oe-PELI1) was determined by western blot. **F** Colony formation analysis showed that oe-PELI1 could partially reverse the miR-30c-5p-mediated proliferation inhibition of PTC cells (n = 3). **G** Transwell analysis showed that oe-PELI1 significantly alleviated miR-30c-5p-mediated migration inhibition of PTC cells (n = 3). **H** p-AKT, AKT, Ki-67 and MMP2 protein levels were determined by western blot in each group. *P < 0.05, **P < 0.01, ***P < 0.001

Then, we investigated the biological role of miR-30c-5p in vitro. CCK-8 assay showed that PTC cells overexpressing miR-30c-5p had markedly lower proliferation ability than the cells transfected with miR-NC (Fig. 5A). Moreover, the colony number of cells with miR-30c-5p was significantly reduced compared to negative control (Fig. 5B). Furthermore, Transwell and wound healing assays showed that miR-30c-5p transfection led to a weakened capacity of cells to migrate (Fig. 5C and D).

Considering PELI1 is a downstream target of miR-30c-5p, we determined whether the restoration of PELI1 could reverse the miR-30c-5p-mediated inhibition of proliferation and migration in PTC cells. The protein expression of PELI1 in miR-30c-5p-overexpressing PTC cells was increased by transfection of a PELI1 cDNA (Fig. 5E). Our data showed that the overexpression of PELI1 (oe-PELI1) significantly impaired the effect of miR-30c-5p, thus remarkably increasing the proliferation and migration in miR-30c-5p-overexpressing PTC cells (Fig. 5Fand G) accompanied by the upregulation of p-AKT, Ki-67 and MMP2 protein (Fig. 5H). Therefore, PELI1 is essential for the miR-30c-5p-mediated inhibition of PTC cell proliferation and migration.

### EVs derived from miR-30c-5p-modified hUCMSC (miR-30c-5p-EVs) can effectively inhibit PELI1 expression and suppress PTC progression

Recently, EVs (nanoscale membranous vesicles), especially those derived from MSCs, are considered as novel and effective drug vehicles for delivering exogenous nucleic acid into cancer cells [29, 30], thereby possibly enabling a clinical relevant gene-based theraoeutic strategy for cancer. To promote the clinical transformation of miR-30c-5p-PELI1-AKT axis in PTC, we packed miR-30c-5p into hUCMSC-EVs (miR-30c-5p-EVs) and further investigated the effect of miR-30c-5p-EVs on the expression of PELI1 and the progression of PTC.

**Fig. 6:**
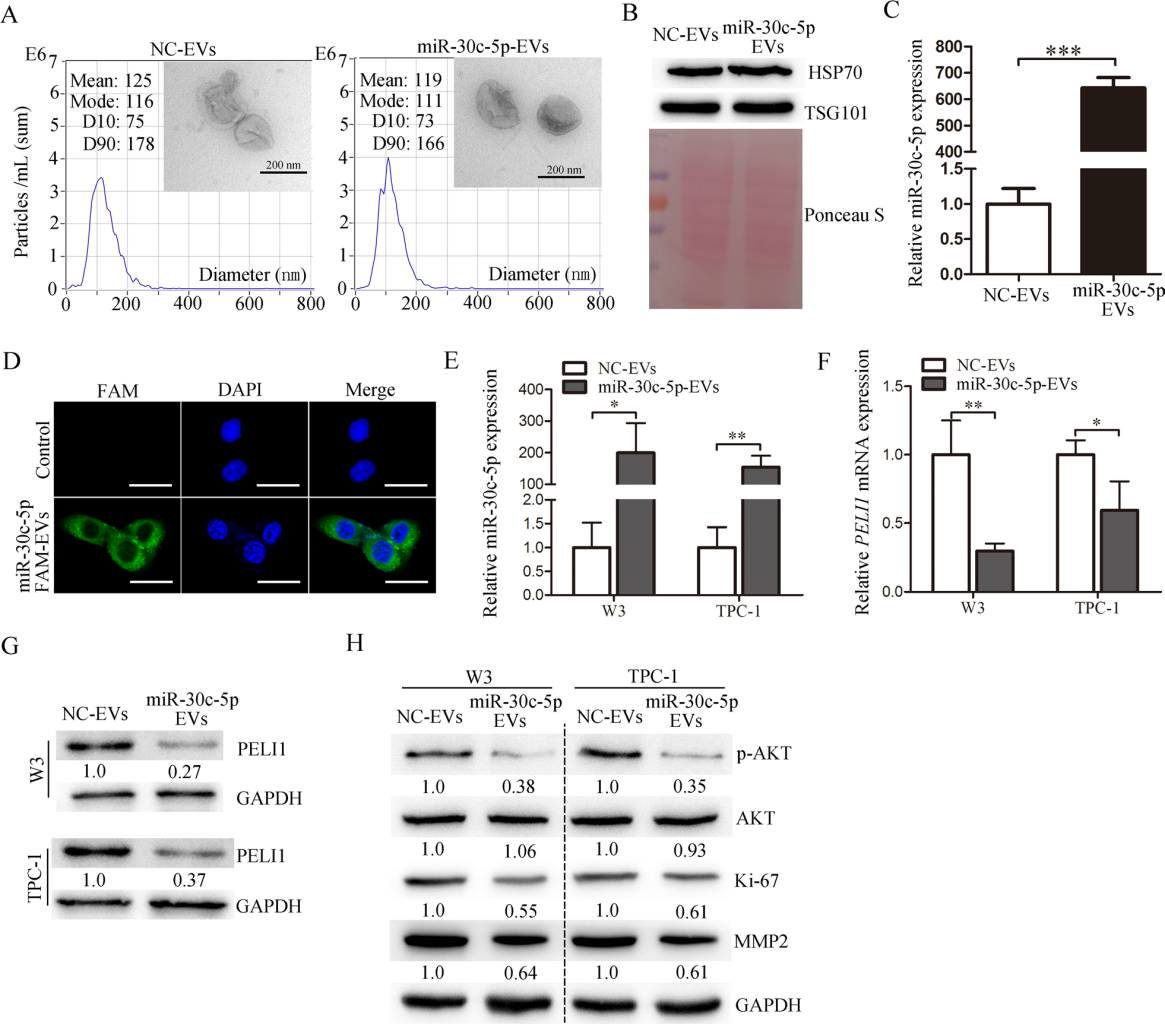
EVs derived from miR-30c-5p-modified hUCMSC (miR-30c-5p-EVs) downregulate PELI1 expression in PTC cells. **A** miR-30c-5p-EVs and NC-EVs were observed under a transmission electron microscope (Scale bars: 200 nm), and the size distributions of these EVs were detected using the Nanoparticle Tracking Analysis. **B** Western blot analysis of TSG101 and HSP70 expression in miR-30c-5p-EVs and NC-EVs. Ponceau S staining served as a loading control. **C** The relative miR-30c-5p levels in miR-30c-5p-EVs and NC-EVs were detected by real-time PCR (n = 3). **D** Fluorescence was evaluated using laser confocal microscopy (Scale bars: 20 μm). **E** PTC cells treated with miR-30c-5p-EVs showed a significantly increased expression of miR-30c-5p in comparison with cells added with NC-EVs (n = 3). **F**, **G** Expression of PELI1 in PTC cells was assessed by real-time PCR (**F**) and Western blot (**G**). **H** p-AKT, AKT, Ki-67 and MMP2 protein levels were determined by western blot in each group. *P < 0.05, **P < 0.01, ***P < 0.001

As shown in Fig. 6A, miR-30c-5p-EVs are round membrane-bound vesicles and have an average diameter of 119 nm with a size distribution of 50–300 nm. Western blot showed that several EV markers including heat shock protein 70 (HSP70) and tumor susceptibility gene 101 (TSG101) [31] are present in these vesicles (Fig. 6B). As expected, a significant increase in miR-30c-5p levels compared to controls (NC-EVs) was observed in miR-30c-5p-EVs (Fig. 6C), confirming that miR-30c-5p was successfully be loaded into hUCMSC-EVs.

Then, we transfected hUCMSCs with FAM-labled-miR-30c-5p, collected the EVs containing FAM-miR-30c-5p (called miR-30c-5p-FAM-EVs), and treated W3 cells with miR-30c-5p-FAM-EVs. Green signals were detected in the cytoplasm of W3 cells exposed to these miR-30c-5p-FAM-EVs under fluorescence microscopy (Fig. 6D), suggesting that miR-30c-5p in miR-30c-5p-EVs could be effectively transferred into PTC cells. Indeed, the miR-30c-5p level was significantly increased in PTC cells treated with miR-30c-5p-EVs compared with that in the NC-EV group (Fig. 6E). Moreover, the mRNA and protein expression of PELI1 were downregulated in PTC cells treated with miR-30c-5p-EVs (Fig. 6F and G). In addition, compared with NC-EV group, miR-30c-5p-EV treatment decreased the protein levels of p-AKT, Ki-67 and MMP2 in PTC cells (Fig. 6H).

**Fig. 7:**
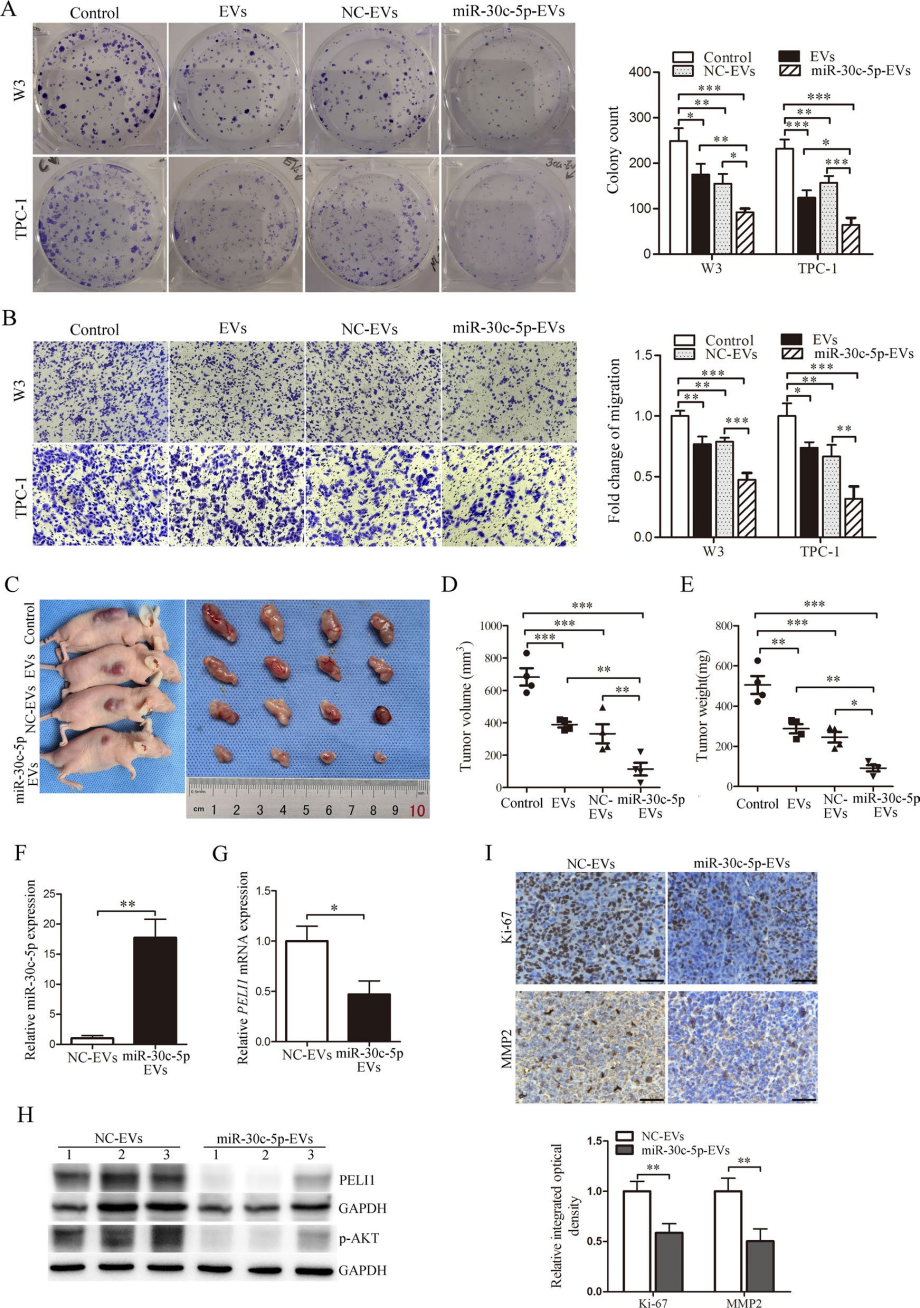
MiR-30c-5p-EVs suppress the progression of PTC in vitro and in vivo. **A** Proliferation of PTC cells in each group was detected using colony formation assay (left panel), and the colony number was counted (right panel; n = 3). **B** The migration properties in each group were determined by using Transwell assay (left panel). Magnification, × 200. Collective analysis of the migration cell number from EVs-treated-PTC cells (right panel; n = 3). **C** Representative images of xenografts in W3 tumour-bearing nude mice at 28 days postimplantation. **D**, **E** Changes in tumor volumes (**D**) and weight (**E**) in each W3 xenograft model group. **F** Changes of miR-30c-5p expression were detected by real-time PCR. **G** The expression of *PELI1* in mice tumor treated with miR-30c-5p-EVs was detected by real-time PCR. **H** Western blot analysis showing the levels of PELI1 and p-AKT in the tumor samples. **I** Ki-67 and MMP2 immunohistochemical staining on tumor sections from the NC-EV and miR-30c-5p-EV group (up panel), and the quantification of Ki-67 and MMP2 expression (down panel). The scale bars: 50 µm. *P < 0.05; **P < 0.01, ***P < 0.001

Then, colony-forming and Transwell assays were performed to evaluate the effect of miR-30c-5p-EVs on PTC carcinogenesis in vitro. As shown in Fig. 7A and B, miR-30c-5p-EV treatment significantly inhibited PTC cell proliferation and migration compared with the NC-EV group. To investigate whether miR-30c-5p-EVs could inhibit PTC cells in vivo, miR-30c-5p-EVs was administered by intra-tumor injection to nude mice bearing W3 cells. As shown in Fig. 7C–E, the volume and weight of the tumor treated with miR-30c-5p-EVs was remarkably decreased compared with the NC-EV group. In view of the fact that MSC-EVs exert various effects on cancer cell [32], we then investigated the effect of naïve hUCMSC-EVs on PTC carcinogenesis, and found that compared with treatment with PBS (Control group), treatment of PTC cells with hUCMSC-EVs resulted in a dramatic reduction in proliferation and migration in vitro (Fig. 7A and B) and tumor volume and weight in vivo (Fig. 7C–E).

Then, the expression of miR-30c-5p and *PELI1* in the tumor tissues were determined using real-time PCR, and we found that compared with the NC-EV group, the expression of miR-30c-5p was significantly upregulated, whereas the level of *PELI1* was downregulated in the miR-30c-5p-EV group (Fig. 7F and G). We performed a western blot analysis of the relative expression of PELI1 and p-AKT in the mice tumor and found that PELI1 and levels of p-AKT were decreased in miR-30c-5p-EV-treated tumors compared with NC-EV-treated tumors (Fig. 7H). In addition, IHC analysis revealed that fewer Ki-67-positive and MMP2-positive cells were observed in the tumor sections from the miR-30c-5p-EV group than in NC-EV group (Fig. 7I). Taken together, these results suggested that EVs from hUCMSCs treated with miR-30c-5p mimics could effectively decrease the PELI1 expression in PTC and suppress PTC progression.

## Discussion

As an E3 ubiquitin ligase, PELI1 contributes to lymphoid and several solid (e.g., lung cancer, breast cancer, and melanoma) tumorigenesis [5–10], which has been emphasized as valuable prognostic biomarker and attractive therapeutic targets of cancer. However, the expression and the biological function of PELI1 in PTC remain unclear. In this study, we presented the first evidence that both mRNA and protein levels of PELI1 widely upregulated in PTC tumor samples. In addition, we found that *PELI1* mRNA overexpression was associated with larger tumor size and lymph node metastasis, which consistent with the results from TCGA database that upregulation of *PELI1* in PTC patients was closely associated with *Ki-67* and lymph node metastasis. These data indicated that PELI1 potentially functions as a prognostic marker in PTC. Ectopic expression of PELI1 promoted PTC cell proliferation and migration, while PELI1 knockdown decimated these cellular behaviors of PTC cells in vitro*.* More importantly, PELI1 knockdown inhibited tumor growth in subcutaneous tumor model accompanied with the decreased expression of Ki-67 and cell metastasis marker MMP2. These data strongly suggested that PELI1 contributed to PTC malignant progression and was therapeutic target for PTC. However, contradictory results regarding PELI1 expression and its roles in esophageal squamous cancer have been described recently [11], perhaps mainly because of differences in tumor types, tumor microenvironments, animal model and so on.

PI3K/AKT pathway is closely associated with THCA development and progression and considered as new therapies for advanced THCA [2, 26, 27]. Combined with KEGG analysis, the positive regulation of p-AKT by PELI1 in PTC cells and animal tumors was verified by using western blotting. These findings were similar to Jeon’ s report that p-AKT was elevated in PELI1-overexpressing A549 and H1299 cells, but decreased in PELI1-depleted A549 and H1299 cells [8]. Additionally, in this study, we found that the inhibition of p-AKT significantly inhibited PELI1-mediated PTC cell proliferation and migration, which further confirmed that PELI1 promoted PTC progression, at least partly, through PI3K/AKT activation. However, the mechanism by which PELI1 activates PI3K/AKT pathway in PTC cells remains to be elucidated. It should be noted that a recent study reported that the PELI1 deficiency in T cells did not affect AKT activation [10]. Thus, the functional roles of PELI1 in PI3K/AKT pathway might be cell type specific.

MiRNAs are thought to primarily down regulate gene expression by binding to 3′UTR of target transcripts, thereby usually be considered as fine-tuners of gene and signaling [12–14]. Until now, no study has investigated the relationship between miRNAs and PELI1 in tumors. Based on bioinformatics analysis, we considered that miR-30c-5p loss might induce PELI1 accumulation in PTC, which was confirmed later by the following evidences: first, an inverse correlation between miR-30c-5p and PELI1 expression was observed in PTC tissues. Second, luciferase activity assays indicated that miR-30c-5p could bind with the 3′-UTR of *PELI1*. Third, miR-30c-5p inversely regulated PELI1 mRNA and protein abundance in PTC cells. Certainly, this study did not rule out that other miRNAs may also be involved in PELI1 regulation, because an mRNA might be targeted simultaneously by many miRNAs [12]. Furthermore, we first disclosed the biological function of miR-30c-5p in PTC. We demonstrated that miR-30c-5p overexpression inhibited PTC cell proliferation and migration in vitro, and these inhibition effects were partly abrogated by PELI1 restoration. These results not only supported the notion that miR-30c-5p is a tumor suppressor [33–37] but also further confirmed the tumor-promoting function of PELI1 in PTC.

Due to poor cellular uptake and degradation after systemic delivery, delivering nucleic acid to tumors has been challenging [38, 39]. Although liposomes and viral-based delivery systems have been assessed, all of these approaches exhibit low efficiency [40]. Using EVs as biological vehicles to deliver tumor suppressor gene for tumor treatment is a novel and promising approach [41, 42]. Especially, MSC is well suited for mass production of EVs that are ideal for gene delivery [43, 44]. Our group and other investigators previously showed that MSC-EVs could effectively carry exogenous nucleic acid to influence the progression of tumor cells [17, 20, 30, 45]. However, miRNA-carrying MSC-EVs strategy for PTC therapy has still not been explored. In this context, we selected hUCMSCs as the source of MSC-derived EVs, as hUCMSCs are better choices of MSCs for clinical application because of its higher cell vitality, higher accessibility, lower senescence, and fewer ethical constrains than other adult counterparts [46, 47]. Our results showed that (1) miR-30c-5p-EVs could effectively delivered miR-30c-5p to PTC cell, and decrease the mRNA and protein expression of PELI1 and inhibited the PI3K/AKT pathway; (2) treatment with miR-30c-5p-EVs inhibited PTC cell proliferation and migration in vitro; and (3) miR-30c-5p-EVs were efficacious against PTC tumor growth in the BALB/c nude mice model, together with the upregulation of miR-30c-5p and significant downregulation of PELI1, p-AKT, Ki-67, and MMP2 expression. Overall, these data provided strong evidence that engineered hUCMSC-EVs delivering miR-30c-5p could effectively inhibit PELI1 expression and tumor progression in PTC, which partly promoted the clinical transformation of miR-30c-5p-PELI1 axis in tumor treatment. More importantly, in present study, we first investigated the effects of naïve hUCMSC-EVs on PTC progression, as MSC-EVs might exert various effects on cancer cell [32]. We found hUCMSC-EVs significantly decreased PTC cell proliferation and migration in vitro and inhibited tumor growth in vivo. These findings together seemed to support the recent notion that MSC-EVs not only are versatile anti-tumor agent delivery platforms, but also can be a cell-free cancer treatment alternative [48]. Further investigation is required to completely elucidate the role and mechanism of hUCMSC-EVs in PTC.

## Conclusions

**Fig. 8:**
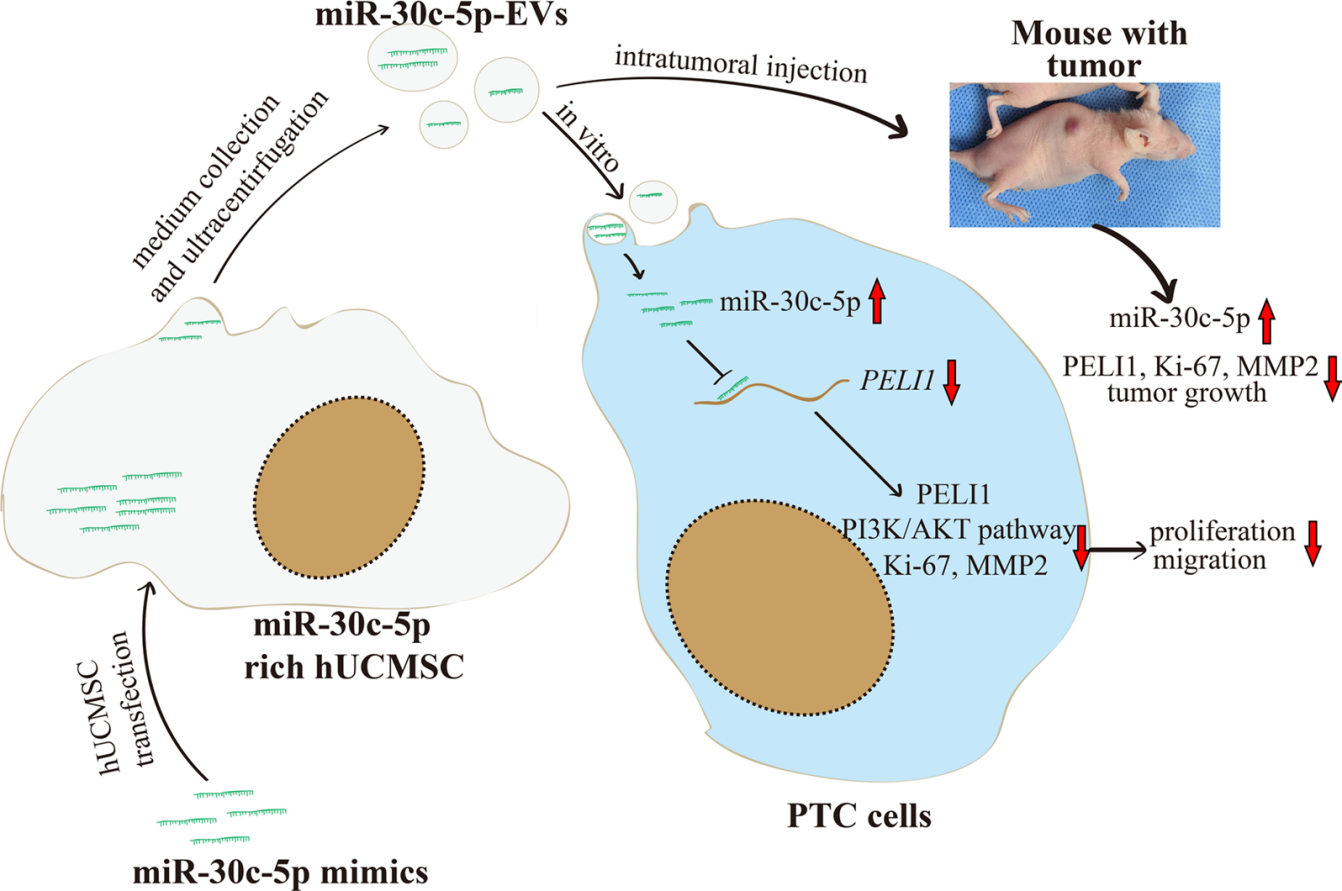
Proposed model of miR-30c-5p-EVs as new avenues for the treatment of PTC cancer. PELI1 was highly expressed in PTC cancer, while miR-30c-5p was poorly expressed. MiR-30c-5p could inhibit PTC cell proliferation and migration by negatively mediating the expression of the PELI1. MiR-30c-5p-EVs could significantly downregulate PELI1 expression and suppress the progression of PTC in vitro and in *vivo*, concomitant with reduced p-AKT, Ki-67 and MMP-2 expression

To our knowledge, the present study first revealed that miR-30c-5p loss-induced PELI1 accumulation regulates cell proliferation and migration via activating PI3K/AKT pathway in PTC. Moreover, miR-30c-5p EVs could effectively downregulate PELI1 expression and suppresses the progression of PTC (Fig. 8). These findings may offer potentially novel MSC-EVs-based therapeutic strategies for patients with PTC or other miR-30c-5p-PELI1 abnormal diseases.

## Supplementary Information

**Additional file 1:** Genes derived from TCGA THCA databases, whose pearson correlation coefficient value with *PELI1* are >0.5.

**Additional file 2:** The co-expressed genes that were associated with PI3K-AKT pathway.

**Additional file 3:** MiRNAs that might target *PELI1* from miRanda, PicTar and TargetScan.

## Data Availability

All data generated or analyzed during this study are included in this published article.

## Abbreviations

PELI1: Pellino-1
THCA: Thyroid cancer
PTC: Papillary thyroid cancer
MMP-2: Metallopeptidase 2
EVs: Extracellular vesicles
HUCMSC: Human umbilical cord mesenchymal stem cells
IHC: Immunohistochemical assay
HSP70: Heat shock protein 70
TSG101: Tumor susceptibility gene 101
TCGA: The cancer genome atlas
