# Supplementary figures and images for: MiR-30c-5p loss-induced PELI1 accumulation regulates cell proliferation and migration via activating PI3K/AKT pathway in papillary thyroid carcinoma
Source: J Transl Med. 2022 Jan 6;20:20. doi: 10.1186/s12967-021-03226-1 (PMC8740468; doi:10.1186/s12967-021-03226-1)

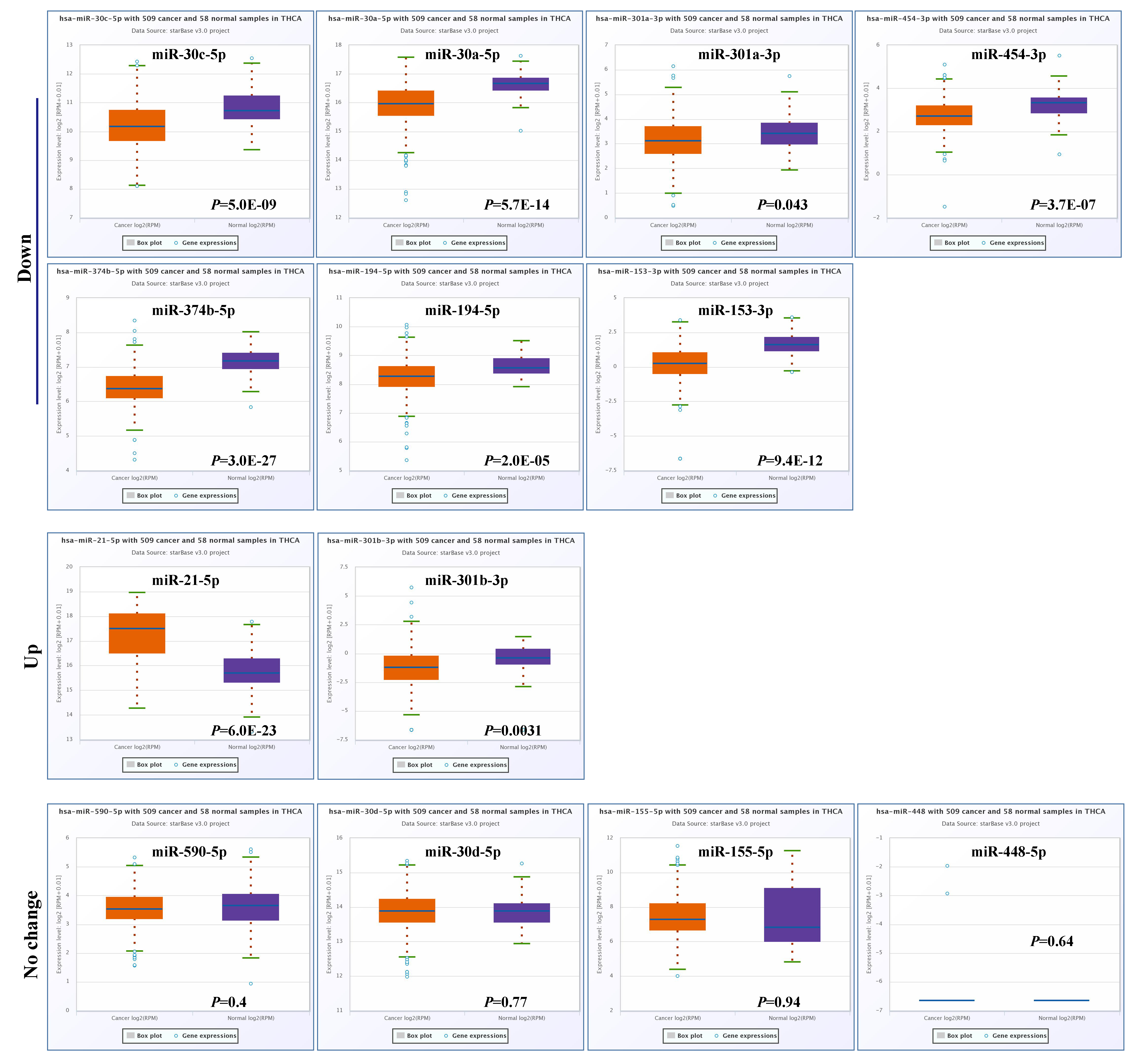

Supplement: Supplementary file 3 — Additional file 3: MiRNAs that might target PELI1 from miRanda, PicTar and TargetScan. [file 12967_2021_3226_MOESM3_ESM.tif]
